# The Soluble Adenylyl Cyclase Inhibitor LRE1 Prevents Hepatic Ischemia/Reperfusion Damage Through Improvement of Mitochondrial Function

**DOI:** 10.3390/ijms21144896

**Published:** 2020-07-11

**Authors:** João S. Teodoro, João A. Amorim, Ivo F. Machado, Ana C. Castela, Clemens Steegborn, David A. Sinclair, Anabela P. Rolo, Carlos M. Palmeira

**Affiliations:** 1 Center for Neurosciences and Cell Biology of the University of Coimbra, 3004-504 Coimbra, Portugal; Joao_Amorim@hms.harvard.edu (J.A.A.); ivofmachado@gmail.com (I.F.M.); catycastela@hotmail.com (A.C.C.); anpiro@ci.uc.pt (A.P.R.); 2 Department of Life Sciences of the University of Coimbra, 3000-456 Coimbra, Portugal; 3 IIIUC—Institute of Interdisciplinary Research of the University of Coimbra, 3030-789 Coimbra, Portugal; 4 Department of Genetics, Blavatnik Institute, Paul F. Glenn Center for the Biology of Aging, Harvard Medical School, Boston, MA 02115, USA; david_sinclair@hms.harvard.edu; 5 Department of Biochemistry, University of Bayreuth, 95440 Bayreuth, Germany; clemens.steegborn@uni-bayreuth.de; 6 Laboratory for Ageing Research, Department of Pharmacology, School of Medical Sciences, The University of New South Wales, Sydney 2052, Australia

**Keywords:** mitochondria, ischemia/reperfusion, liver, soluble adenylyl cyclase, LRE1, sirtuin 3

## Abstract

Hepatic ischemia/reperfusion (I/R) injury is a leading cause of organ dysfunction and failure in numerous pathological and surgical settings. At the core of this issue lies mitochondrial dysfunction. Hence, strategies that prime mitochondria towards damage resilience might prove applicable in a clinical setting. A promising approach has been to induce a mitohormetic response, removing less capable organelles, and replacing them with more competent ones, in preparation for an insult. Recently, a soluble form of adenylyl cyclase (sAC) has been shown to exist within mitochondria, the activation of which improved mitochondrial function. Here, we sought to understand if inhibiting mitochondrial sAC would elicit mitohormesis and protect the liver from I/R injury. Wistar male rats were pretreated with LRE1, a specific sAC inhibitor, prior to the induction of hepatic I/R injury, after which mitochondria were collected and their metabolic function was assessed. We find LRE1 to be an effective inducer of a mitohormetic response based on all parameters tested, a phenomenon that appears to require the activity of the NAD^+^-dependent sirtuin deacylase (SirT3) and the subsequent deacetylation of mitochondrial proteins. We conclude that LRE1 pretreatment leads to a mitohormetic response that protects mitochondrial function during I/R injury.

## 1. Introduction

Ischemia/reperfusion (I/R) injury is common but multifactorial event in many surgical situations and, in some cases, in pathological conditions. It occurs when blood flow is restricted to at least part of an organ and is then restored. I/R is characterized by cellular injury, a major decrease in mitochondrial function, and a concomitant decrease in both energetic homeostasis and metabolic competency.

Hypoxic conditions that occur during ischemia cause a metabolic shift away from mitochondrial oxidative phosphorylation (OXPHOS) towards glycolysis. During reperfusion, cells are further stressed due to a burst in oxygen availability and the rebalancing of pH, among other factors [1,2], leading to a burst in oxidative stress caused by reactive oxygen and nitrogen species (ROS and RNS, respectively) [3,4,5]. Another key initial player is intracellular calcium overload [5] and, later on, tissue inflammation.

Central to I/R injury are mitochondria [6,7,8]. These organelles, which are responsible for over 90% of a cells’ ATP requirements, are highly sensitive to perturbations caused by I/R [9,10,11]. As such, strategies that protect mitochondria from I/R injury might be able to prevent tissue damage and save countless lives [9]. A strategy that has shown great potential in several studies hinges on the concept of mitohormesis [12,13], whereby low levels of injury are actually beneficial because they promote the autophagic removal of susceptible and damaged organelles, which are eventually replenished by newer ones, more capable of dealing with the injury. However, higher doses of insult do not generate a protective response strong enough to deal with the insult, resulting in cellular injury, cell death, and organ dysfunction [14]. One way to achieve a mitohormetic response is to prime mitochondria to deal with an injury using pharmacological or surgical means.

Achieving a mitohormetic response requires the activation of mitodynamic processes, such as fusion/fission of mitochondria, removal of injured units (mitophagy), and repopulation of the cell with newly generated mitochondria (biogenesis). This requires close communication between nuclear and mitochondrial genomes, as well as a tight signaling cascade of events that indicate which units to remove and which to replicate. Furthermore, post-translational modification of mitochondrial proteins by reversible phosphorylation/deacetylation by cAMP-dependent or sirtuin-dependent pathways is also crucial (reviewed in [14]).

cAMP is a ubiquitous second messenger involved in mitochondrial dynamics, metabolic homeostasis, and oxidative stress [15,16]. Ten adenylyl cyclases (AC) have so far been identified, but of great relevance to mitochondrial function was the identification of a soluble form (sAC), present in the mitochondrial matrix, aside from the already known transmembrane version [17,18]. sAC promotes the activation of protein kinase A (PKA), which elevates OXPHOS rates and decreases oxidative stress. There are some unanswered questions about the existence and function of PKA within mitochondria, in particular, its role in potentially regulating mitochondrial cAMP [19]. Regardless, the effects of cAMP in mitochondria are indisputable, and the increased mitochondrial function caused by activation of sAC is well accepted.

One of the effects of cAMP production in mitochondria is the activation of SirT3, a member of the sirtuin family of NAD^+^-dependent lysine deacylases/deacetylases [20]. SirT3 is the only native mitochondrial member of this family of enzymes whose activity has been directly linked to elevated mitochondrial function [21,22]. Interestingly, SirT3 is a known inducer of mitohormesis [23,24] and is vital for ROS detoxification [23], raising the possibility that sAC’s effects on mitochondrial function might work via SirT3.

Based on these findings, we postulated that by inhibiting sAC with a specific inhibitor (LRE1), a hormetic response would be induced that would, by activating SirT3, prime hepatic mitochondria to be more resilient to injury caused by a deleterious event such as I/R.

## 2. Results

### 2.1. Hepatic Mitochondrial Bioenergetics

**Figure 1:**
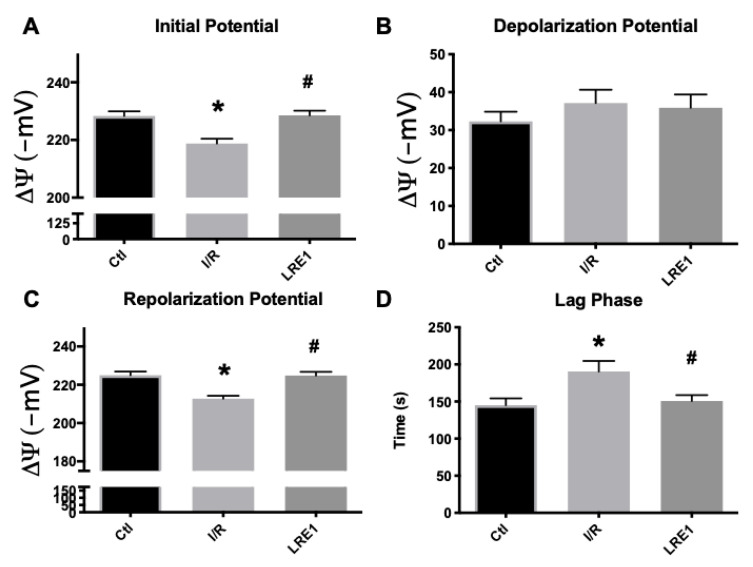
Mitochondrial membrane initial potential (**A**), ADP-driven depolarization potential (**B**), repolarization potential (**C**) and lag phase time (**D**) as evaluated by tetraphenylphosphonium ion (TPP^+^) fluctuations and recorded with a TPP^+^-sensitive electrode. Data are means ± SEM of 4 independent experiments. * indicates a statistically significant difference vs. sham operated controls (Ctl); # indicates a statistically significant difference vs. ischemia/reperfusion (I/R).

Data from isolated liver mitochondria from all experimental groups are represented in this section. As can be seen in Figure 1A,C, mitochondrial resting (initial) and repolarization (post-ADP phosphorylation) potentials were significantly diminished by the I/R, as previously reported [25]. LRE1 pretreatment of 30 min with a 1.2 mg/kg dose was sufficient to prevent said decrease, as was the case with the lag phase time (Figure 1D), i.e., the time required to phosphorylate a set amount of ADP and return the potential to a steady value. However, there were no differences, for any groups, in the depolarization potential (Figure 1B), i.e., the maximal decrease in potential as caused by a fixed (200 nmol) amount of ADP.

**Figure 2:**
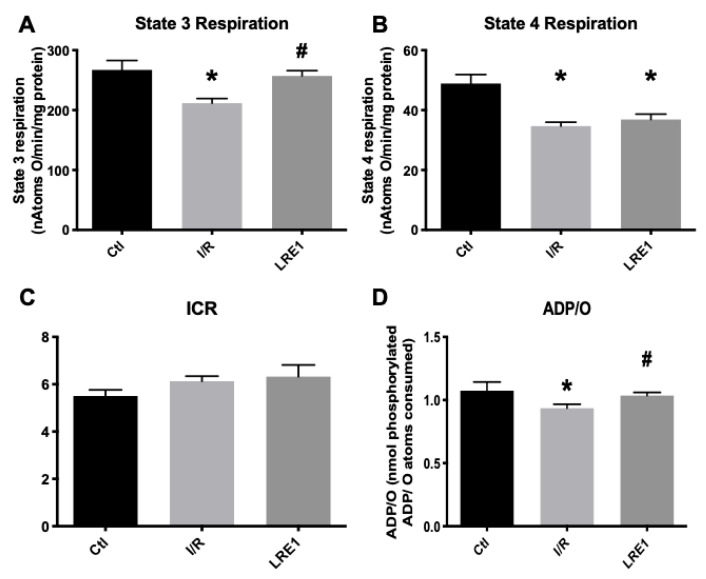
Mitochondrial oxygen consumption parameters (ADP-driven State 3 respiration, **A**; repolarization respiration State 4, **B**) and resulting ratios (Respiratory Control Index, ICR, **C** and; ADP/O, **D**), as evaluated by O_2_ consumption and recorded with a Clark-type electrode. Data are means ± SEM of 4 independent experiments. * indicates a statistically significant difference vs. Ctl; # indicates a statistically significant difference vs. I/R.

Furthermore, simultaneous data on mitochondrial respiration, as evaluated with a Clark-type electrode, were recorded on the same samples as above. The obtained data are represented in Figure 2. In accordance with Figure 1, state 3 respiration (ADP-driven respiration) was affected by I/R injury, as was the ADP/O ratio, i.e., the ratio indicating how much ADP can be phosphorylated by the same amount of oxygen consumed. Predictably, LRE1 pretreatment recovered both state 3 respiration and ADP/O ratios (Figure 2A,D, respectively), but not state 4 respiration, i.e., post-ADP phosphorylation respiration (Figure 2B), a parameter that was also affected in the I/R group. Interestingly, the ICR ratio (respiratory control index) was not altered in any experimental group.

**Figure 3:**
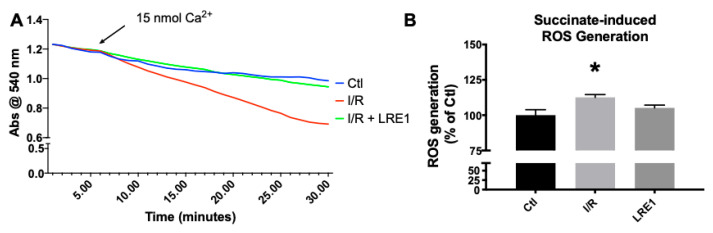
Mitochondrial swelling recording (**A**) and reactive oxygen species (ROS) generation (**B**). Swelling was recorded by following absorbance of light at 540 nm after a Ca^2+^ challenge, while ROS was evaluated by following 2′,7′-dichlorodihydrofluorescein diacetate (H2DCFDA) fluorescence for 10 min. For panel A, lines indicate a representative recording of all of the assays, while panel B represents means ± SEM of 4 independent experiments. * indicates a statistically significant difference vs. Ctl.

All these data from both Figure 1 and Figure 2 indicate that LRE1 was a competent, albeit incomplete, effector of mitochondrial damage prevention through sAC inhibition. Further ex vivo testing of these mitochondrial preparations was performed. The data are represented in Figure 3 (mitochondrial swelling recording and ROS generation).

As can be seen in Figure 3A, mitochondrial resilience to mPT induction by means of a calcium challenge was significantly affected by I/R, a clear indicator of a reduction in available membrane potential (as seen in Figure 1) and overall poorer bioenergetic status. Once more, LRE1 was a competent preventer of I/R-driven alterations. Statistical analysis indicates that the I/R group was significantly lower than the other two groups at the 22 min mark. As for ROS generation, I/R drove ROS generation up in isolated liver mitochondria, an effect that was prevented by LRE1 pretreatment (Figure 3B).

**Figure 4:**
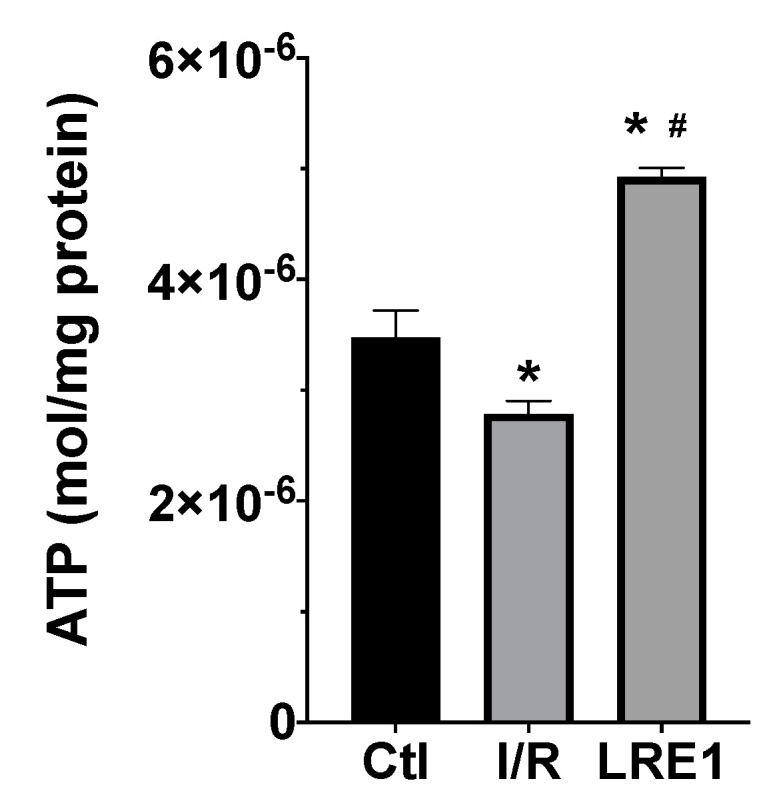
Liver mitochondria ATP content as evaluated with a bioluminescent assay. Data are means ± SEM of 4 independent experiments. * indicates a statistically significant difference vs. Ctl; # indicates a statistically significant difference vs. I/R.

Furthermore, we evaluated ATP levels within mitochondria subjected to I/R with and without LRE1 pretreatment. ATP quantification is represented in Figure 4.

As expected, I/R caused a significant reduction in mitochondrial ATP content. However, LRE1 pretreatment tremendously elevated ATP levels when compared both with the Ctl and I/R groups, a clear marker of the previously shown data, indicating that LRE1 pretreatment had ameliorated I/R deleterious events on hepatic mitochondrial function, even when compared with the Ctl group.

In conclusion, LRE1 pretreatment was capable of preventing most of the alterations to mitochondrial bioenergetics and evaluated functional parameters.

### 2.2. Gene Expression and Protein Content

**Figure 5:**
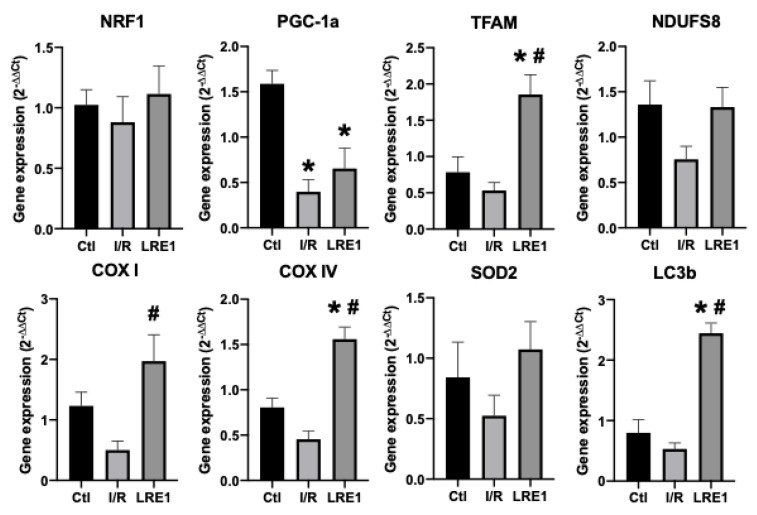
Liver gene expression evaluation by semi-qPCR. Data are means ± SEM of 4 independent experiments. *NRF1*, nuclear respiratory factor 1; *PGC-1a*, peroxisome proliferator-activated receptor gamma, coactivator 1 alpha; *TFAM*, mitochondrial transcription factor A; *NDUFS8*, NADH dehydrogenase iron–sulfur protein 8, mitochondrial; *COX I*, cytochrome c oxidase subunit I; *COX IV*, cytochrome c oxidase, subunit IV; *SOD2*, superoxide dismutase 2, mitochondrial; *LC3b*, microtubule-associated proteins 1A/1B light chain 3B. * indicates a statistically significant difference vs. Ctl; # indicates a statistically significant difference vs. I/R.

**Figure 6:**
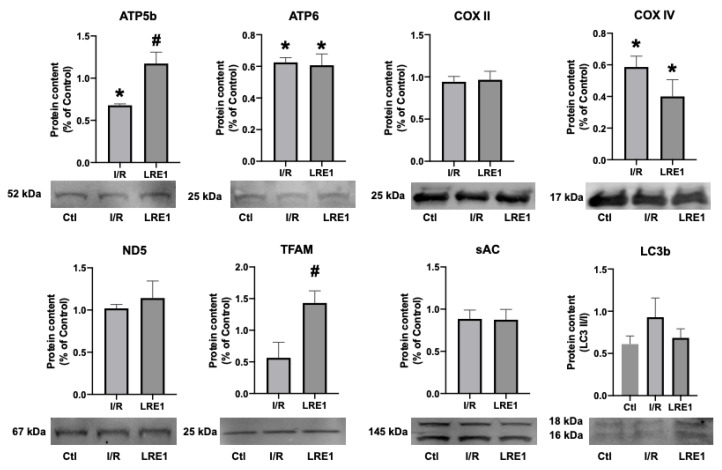
Hepatic mitochondrial protein content quantification by Western blot. Data are means ± SEM of 4 independent experiments. ATP5b, ATP synthase F1 subunit beta, mitochondrial; ATP6, ATP synthase F_O_ subunit 6, mitochondrial; COX II, cytochrome c oxidase subunit II; COX IV, cytochrome c oxidase, subunit IV; ND5, NADH-ubiquinone oxidoreductase chain 5; TFAM, mitochondrial transcription factor A; sAC, soluble adenylyl cyclase; LC3b, microtubule-associated proteins 1A/1B light chain 3B. * indicates a statistically significant difference vs. Ctl; # indicates a statistically significant difference vs. I/R.

To further evaluate the protective effects of sAC inhibition by LRE1 pretreatment on I/R injury, tissue samples and mitochondrial preparations were processed for gene expression and protein content analysis, respectively. Figure 5 and Figure 6 illustrate the results obtained.

As can be seen in FLRE1 pretreatment significantly elevated the gene expression of the genes coding for mitochondrial complex IV (*COX*) subunits I and IV, the mitochondrial transcription factor A (*TFAM*), and microtubule-associated proteins 1A/1B light chain 3B (*LC3b*), a regulator of mitophagy. Conversely, gene expression for the peroxisome proliferator-associated receptor gamma coactivator 1α (*PGC-1a*) expression was diminished by I/R injury and not rescued by LRE1 pretreatment. The elevation of *TFAM* and *COX* subunits expression indicates that the LRE1 pretreatment initiated mitochondrial biogenesis. The lack of effect on *PGC-1α* and *NRF1* genes expression might be due to a timing effect, since these players are involved in the early phases of mitochondrial biogenesis. Despite a clear trend towards the recovery of *SOD2* levels, no statistical effect was observed at this time point. However, the elevation in *LC3b* expression clearly indicates an increase in autophagic signaling that is known to be required for mitochondrial biogenesis and an improvement of mitochondrial function.

**Figure 7:**
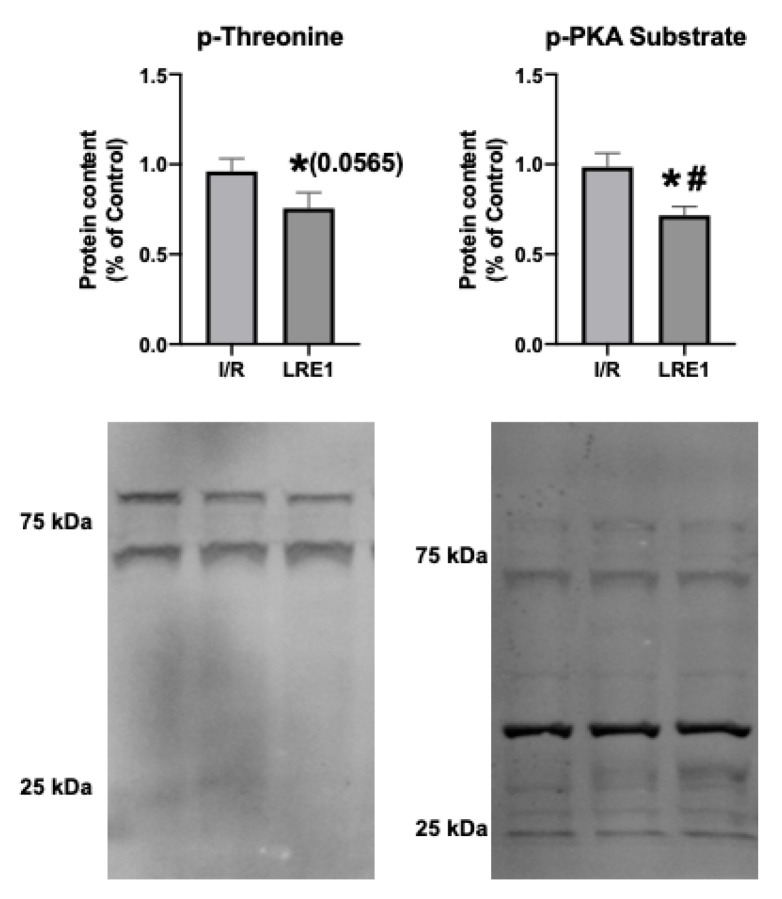
Hepatic mitochondrial protein phosphorylation content quantification by Western blot. Data are means ± SEM of 4 independent experiments. p-threonine, phosphorylated threonine residues; p-PKA substrate, phosphorylated PKA substrate. * indicates a statistically significant difference vs. Ctl; # indicates a statistically significant difference vs. I/R.

Next, we examined targets of sAC activity, namely, the commonly phosphorylated residues of mitochondrial proteins. cAMP generated by sAC within mitochondria has been shown to lead to the activation of PKA, thus contributing to the phosphorylation of several proteins within mitochondria [26,27]. Figure 7 shows the results obtained, where it is visible that LRE1 pretreatment led to the decrease in phosphorylation of total mitochondrial protein extracts.

**Figure 8:**
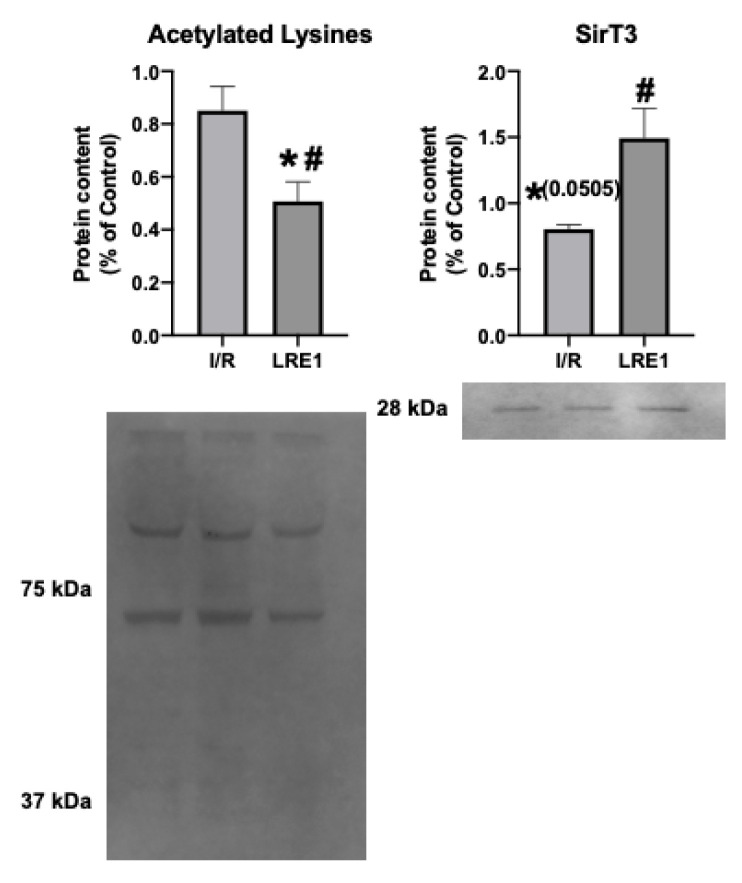
Hepatic mitochondrial protein acetylation status and NAD^+^-dependent deacetylase sirtuin 3 (SirT3) quantification by Western blot. Data are means ± SEM of 4 independent experiments. SirT3, Sirtuin 3. * indicates a statistically significant difference vs. Ctl; # indicates a statistically significant difference vs. I/R.

A known target of cAMP is the mitochondrial matrix NAD^+^-dependent deacetylase sirtuin 3 (SirT3). SirT3 is a known positive effector of mitochondrial function, and thus insight into its levels and activity could help explain the effects of LRE1. In fact, SirT3 levels have been negatively correlated with elevated cAMP levels [26,27]. As such, we looked into the mitochondrial SirT3 content and mitochondrial acetylation levels. These data are presented in Figure 8.

The data from Figure 8 show that SirT3 levels trended downward in response to I/R, while LRE1 pretreatment was sufficient to prevent SirT3 content reduction and, concomitantly, led to the reduction in the presence of acetylated lysine residues in mitochondrial preparations. Since the presence of acetylated lysines in mitochondria is correlated with a decrease in mitochondrial OXPHOS capacity [26,27], it is likely that the protection of mitochondrial function provided by LRE1 pretreatment against I/R injury is linked to the activation of SirT3 and an increase mitochondrial function and ATP that prevents mitochondrial dysfunction and cell death in response to I/R injury.

## 3. Discussion

Ischemia/reperfusion (I/R) injury is a common phenomenon in various surgical settings, stroke, and chronic diseases such as cardiovascular and cerebrovascular diseases. The removal and restoration of blood flow is a potent inducer of cellular oxidative damage, organelle dysfunction, cellular death, and ultimately organ failure if enough widespread damage occurs [28]. Novel avenues of preventing this damage could be the difference between life and death. Given the central role of mitochondria in I/R, if these strategies are able to prevent mitochondrial dysfunction, then there is a real potential to these therapies. As such, exploring the mitohormetic capacity is a novel avenue to numerous diseases where mitochondrial function is involved [14,29].

A critical regulator of mitochondrial function is cAMP. This ubiquitous second messenger is produced by the adenylyl cyclase (AC) family of enzymes, of which there are numerous members. Of great interest to mitochondrial biology (given their existence in these organelles) are: (1) a transmembrane AC, bound to the outer membrane which produces cAMP that does not permeate the mitochondrial membrane and thus does not affect OXPHOS and other matrix biochemical reactions [30] and (2) a soluble, free form (sAC) present in the matrix that produces virtually all of the cAMP having effects in mitochondria [31]. In response to carbon dioxide, sAC is activated and generates cAMP that ultimately leads to an increase in mitochondrial function, i.e., greater rates of OXPHOS function that ultimately lead to higher levels of the generated ATP [15,32]. Given this, our rationale was that if we can prime mitochondria through a mitohormetic effect to protect them against I/R injury, then the complications associated with this phenomenon could be better tolerated or downright prevented. This reasoning was already hinted at by a sAC knockout mouse model, in which, sAC removal dramatically elevated OXPHOS components [33].

In this study, we directly targeted mitochondrial sAC with a novel compound called LRE1 [34], which is known to allosterically inhibit sAC by binding to the same site as bicarbonate, thereby preventing its activation and reducing cAMP generation. In this and other studies, LRE1 treatment was accompanied by a reduction in mitochondrial OXPHOS respiratory chain complex IV cytochrome *c* oxidase (COX) activity [34]. We hypothesized that pretreatment with LRE1 via its direct injection into the portal vein of animals about to experience surgery-induced I/R, would inhibit mitochondrial sAC and elevate mitochondrial capacity via mitohormesis.

Our data are consistent with this hypothesis. Most of the hepatic mitochondrial parameters we evaluated were reversed to sham-operated control values, despite an I/R event. Mitochondrial membrane potential (ΔΨ) was also maintained, as well as the efficiency of the OXPHOS system, due to the prevention of lag phase elevation (Figure 1). Similarly, the maintenance of the ADP/O ratio and elevated state 3 respiration rates indicated a highly competent OXPHOS system post-I/R (Figure 2). ATP was also dramatically elevated, and as one would expect, mitochondrial function was improved.

Mitochondrial calcium tolerance capacity, as evaluated by swelling induction in response to free Ca^2+^ ions, was retained by LRE1 pretreatment, as well as prevention of oxidative stress as measured by H_2_O_2_ generation in isolated mitochondria (Figure 3). Because both Ca^2+^ mitochondrial metabolism and oxidative stress are hallmarks of I/R injury and both have been involved in mitochondrial sAC-cAMP biology [32,33], it is reasonable to conclude that LRE1 pretreatment induced a mitohormetic response that elevated calcium tolerance and diminished ROS.

To further test our hypothesis, we next examined the expression of key mitochondrial biogenesis genes and genes coding for proteins of the respiratory chain and found that the *TFAM* genes, as well as genes encoding for cytochrome *c* oxidase (*COX*) subunits I and IV, were elevated by LRE1 pretreatment when compared with the I/R group (Figure 4). Interestingly, the gene coding for the master regulator of mitochondrial biogenesis, *PGC-1α*, was not elevated by LRE1 pretreatment. Since PGC-1α is the initial player in mitochondrial biogenesis [35], we attribute this to *PGC-1α* gene activation occurring prior to the time point analyzed. The expression of the *SOD2* gene showed a clear trend towards recovery, and given our ROS generation data (Figure 3), we strongly suspect that it is once more a time point issue. Finally, since the gene coding for *LC3b*, a key player in autophagic processes (which also includes mitophagy [36]) was significantly elevated by LRE1 pretreatment, we conclude that the cellular benefits during I/R injury were likely mediated by mitohormesis-induced mitophagy.

We also hypothesized that PKA activity was reduced by LRE1, with a concomitant reduction of cAMP generation. In Figure 6, we show that LRE1 pretreatment significantly downregulated PKA activation, as indicated by a reduction in p-threonine and p-Serine signals, two main targets of PKA [37,38]. Interestingly, despite claims in the literature that SirT3 is positively regulated by the sAC/cAMP/PKA axis [20,39], we found that SirT3 protein content is elevated by LRE1 pretreatment and is associated with a decrease in the acetylation levels of mitochondrial proteins. This finding helps to explain the improvement of mitochondrial function in LRE1-pretreated hepatic mitochondria post-I/R.

In conclusion, our data support a model in which inhibition of sAC by LRE1 induces a mitohormetic response that protects mitochondria from a large insult such as the one caused by I/R. Future work may include the testing of LRE1 in other injuries relevant to human health such as stroke, transplantation or acute kidney injury.

## 4. Materials and Methods

### 4.1. Materials

Except when specifically indicated, all reagents were purchased from Sigma-Aldrich (St. Louis, MO, USA) and were of the highest grade of purity commercially available.

LRE1 was dissolved in DMSO and originated from Nanosyn (Santa Clara, CA, USA), and its identification and specificity was reported in [40].

### 4.2. Animals

12 male Wistar rats (aged 12 weeks, Charles River Laboratories (Wilmington, MA, USA) were housed under standard conditions (12 h light/dark cycles, with ambient temperature and humidity control) and had free access to rodent chow and acidified water. Animals were allowed to acclimatize for 1 week before experimental procedures. All studies were conducted in accordance with the principles and procedures outlined as “3Rs” in the guidelines of EU (1986/609/EEC and 2010/63/EU), FELASA and the National Centre for the 3Rs (the ARRIVE) and were approved by the Animal Care Committee of the Center for Neurosciences and Cell Biology of Coimbra. We also applied the principles of the ARRIVE guideline for data management and interpretation, and all efforts were made to minimize the number of animals used and their suffering, as standard [41]. This experimental project was approved by the CNC ORBEA (President: Dr. Catarina Oliveira), and was given the reference number ORBEA_150_2016_04112016 on 4 November 2016.

### 4.3. Surgical Induction of Ischemia/Reperfusion

Animals were randomly divided into 3 experimental groups: (a) sham operated controls, Ctl; (b) hepatic ischemia/reperfusion (I/R); and c) hepatic I/R but with a 30 min pretreatment with LRE1 1.2 mg/kg injected in the hepatic artery (LRE1). LRE1 dosage was calculated from a previous work [34]. All animals were anesthetized with a cocktail of ketamine/chlorpromazine (both 50 mg/kg).

A model of partial hepatic ischemia (70%) was used to prevent mesenteric venous congestion by allowing decompression of portal flow through the right and caudate lobes, as previously reported [42]. After being subjected to a midline laparotomy, the hepatic artery and portal vein to the left and median liver lobes were clamped for 90 min, after which a reperfusion phase (lasting 12 h) was initiated by the removal of the clamps.

### 4.4. Mitochondrial Isolation

After the reperfusion period, animals were sacrificed by cervical dislocation and liver mitochondria were isolated using a standard isolation protocol [43], with modifications [44]. All procedures were conducted on ice or ice-cold equipment to prevent sample degradation. Briefly, livers were excised from the organisms and thinly minced with scissors in homogenization buffer (250 mM sucrose, 0.5 mM EGTA, 10 mM HEPES, 0.1% fat-free bovine serum albumin, and BSA; pH 7.4). Medium was decanted and replaced twice to remove the maximal amount of blood possible. Tissue pieces were then homogenized with a Potter-Elvehjem tissue homogenizer. Liver homogenate was then decanted into centrifuge tubes and spun at 1000× *g* for 10 min at 4 °C. Supernatant was decanted into new tubes and was again spun for 10 min at 4 °C, but at 10,000× *g*. The supernatant was aspirated and the mitochondrial pellet was resuspended in fresh homogenization buffer, after which the previous centrifugation cycle was performed again. The supernatant was aspirated and the pellet was resuspended in washing buffer (250 mM sucrose and 10 mM HEPES; pH 7.4). Samples were again immediately spun in the same conditions as mentioned above, and this last step was performed twice to obtain an ultrapure mitochondrial pellet. Mitochondrial protein content was quantified by the Biuret method calibrated with BSA [45].

### 4.5. Mitochondrial Oxygen Consumption Evaluation

Mitochondrial oxygen consumption was polarographically determined using a Clark-type electrode, as previously described [46]. Reactions were conducted at 25 °C in a water bath-jacketed chamber with magnetic stirring. Mitochondria (1 mg) were placed in 1.3 mL of standard mitochondrial respiratory buffer (130 mM sucrose, 50 mM KCl, 5 mM MgCl_2_, 5 mM KH_2_PO_4_, 50 µM EDTA, and 5 mM HEPES; pH 7.4) supplemented with 2 µM rotenone to prevent respiratory chain Complex I contribution and to prevent retrograde electronic flow. Mitochondria were energized with 5 mM succinate, and state 3 respiration was initiated by the addition of 200 nmol ADP. State 3, state 4, Respiratory Control Index, and ADP/O ratios were calculated according to a previous work [43,47].

### 4.6. Mitochondrial Membrane Potential (ΔΨ) Measurement

ΔΨ was estimated using an ion-selective electrode that evaluates the intercompartmental distribution of the tetraphenylphosphonium ion (TPP^+^), a highly soluble ion that freely traverses the inner mitochondrial membrane in direct accordance to ΔΨ, as previously reported [48]. Voltage response of the TPP^+^-selective electrode to the logarithmic TPP^+^ concentration was linear with a slope of 59 ± 1, in accordance with the Nernst equation. These assays were simultaneously performed with the oxygen consumption ones, as described above. A matrix volume of 1.1 µL/mg protein was assumed.

### 4.7. Mitochondrial Permeability Transition Recording

Mitochondrial swelling was evaluated by changes in light scattering as spectrophotometrically measured at 540 nm, in a water bath-jacketed Helios Alpha Spectrophotometer (Thermo Electron Corporation, Waltham, MA, USA) [49]. Reactions were performed at 25 °C, and recording was started by the addition of 1 mg of mitochondrial preparation to 2 mL of swelling medium (200 mM sucrose, 10 mM HEPES, 1 mM KH_2_PO_4_, and 10 µM EGTA; pH 7.4) supplemented with 2 µM rotenone and 5 mM succinate. A negative control reaction was also supplemented with 1 µM cyclosporin A, a known mitochondrial permeability transition inhibitor. After 5 min of basal absorbance recording, 15 nmol Ca^2+^ was added to all reactions and absorbance recording was performed for an additional 25 min.

### 4.8. Mitochondrial Reactive Oxygen Species (ROS) Generation Quantification

ROS generation was fluorometrically determined using a Victor3 plate reader (Perkin-Elmer, Waltham, MA, USA), by recording the fluorescence emitted by 2′,7′-dichlorodihydrofluorescein diacetate (H_2_DCFDA) at 538 nm after excitation at 485 nm [50]. In brief, 1 mg of isolated mitochondria were incubated in standard respiratory medium (as above) for 30 min, with 5 mM succinate and 50 µM H_2_DCFDA (in DMSO), at 37 °C. After 3 min centrifugation at 10,000× *g* to pellet mitochondria, medium was refreshed to remove excess fluorescent probe, after which, 200 µL medium (corresponding to 0.2 mg) was loaded into 96-well plate wells. Fluorescence was recorded for 15 min to calculate a rate of ROS generation. Data are expressed as arbitrary relative fluorescence units (RFUs).

### 4.9. Mitochondrial ATP Content Evaluation

ATP levels were quantified by use of an ATP Kit (Sigma-Aldrich), according to manufacturer’s instructions. Briefly, ATP was extracted by adding 25 µL 2.5 M KOH to 1 mg of mitochondria. Samples were vortexed for a few seconds and centrifuged at 4 °C and 18,000× *g* for 10 min. Supernatants were collected and 100–150 µL of 1 M K_2_HPO_4_ was added, in an amount sufficient for pH neutralization. Samples were then immediately placed on liquid N_2_ and stored at −80 °C for later use. On assay day, ATP was quantified with the bioluminescent kit in a Victor3 plate reader.

### 4.10. qPCR

RNA extraction followed a standard protocol [51]. Briefly, liquid N_2_ flash-frozen mitochondria were thawed in guanidinium thiocyanate (Tri Reagent, Sigma-Aldrich) and mixed with 0.2 volumes of pure chloroform. After a 10 min centrifugation at 4 °C and 12,000× *g*, the upper translucid phase containing RNA was pipetted to a RNAase-free tube, and a clean-up protocol involving 70% ethanol was performed. RNA was desiccated at room temperature and resuspended in molecular grade ultrapure water (Sigma-Aldrich). RNA was quantified using a NanoDrop One instrument (Thermo-Fisher, Waltham, MA, USA) and cDNA was synthesized using the iScript cDNA Synthesis Kit (Bio-Rad, Hercules, CA, USA). cDNA samples were then analyzed by qPCR (CFX 96 Touch, Bio-Rad) using the SsoAdvanced Universal SYBR Green Supermix Kit (Bio-Rad). Gene expression quantification was performed using the 2^−ΔΔ*C*t^ method [15]. Primers used are listed in Table 1. They were previously tested in-house to verify that reaction efficiency was of at least 90%.

### 4.11. Western Blotting

Precisely, 1 mg of flash-frozen mitochondria was lysed in ice-cold RIPA buffer supplemented with a cocktail of protease (Sigma-Aldrich) and phosphatase inhibitors (Pierce, Waltham, MA, USA) and diluted 1:1 in Laemmli buffer with 5% β-mercaptoethanol. Precisely, 50 µg of mitochondrial/Laemmli mix was loaded into homemade SDS-polyacrylamide gels or homemade TGX gels (both from Bio-Rad). Gel percentages depended on target protein size, but roughly targets that weighted over 80 kDa were blotted in 8% gels, while targets below 30 kDa were blotted in 15% gels; intermediate weights were blotted in 10% acrylamide gels. Samples were then electrophoresed at constant voltage and transferred into PVDF membranes by means of a Trans-Blot Turbo apparatus (Bio-Rad). In the case of the TGX gels, these were imaged before transfer in a Bio-Rad’s Gel Doc EZ Imager. After transfer, membranes were blocked with blocking buffer (Thermo-Fisher) for 2 h at room temperature before being cut and exposed overnight at 4 °C to primary antibodies (1:1000), dissolved in TBS-T with 5% blocking solution (listed in Table 2). The following day, membranes were washed 3 times for 20 min each time with TBS-T before incubation for 1 h with secondary antibodies (1:2500). Membranes were again washed 3 times for 20 min each time and incubated for 5–30 min with Qdot 625 streptavidin conjugate substrate (Thermo Fisher) in light-protected tubes (1:2500). Finally, membranes were imaged in the same Gel Doc EZ system as above. Blots were quantified using Bio-Rad’s Image Lab software version 6.0.1.

### 4.12. Statistical Analysis

Represented data are typically means ± SEM of a *n* of 4 different animals per group. Statistical significance was determined using one- or two-way ANOVA tests with a Tukey or Holm-Sidak, post hoc correction ran on GraphPad Prism 8 software for Mac OS (GraphPad, La Jolla, CA, USA). A *p* value ≤ 0.05 was considered statistically significant.

## Figures and Tables

**Table 1:** qPCR primers used.

Primer Name	Sequence	NCBI Nucleotide’s Accession Number
*COX I* Forward	CCA GTA TTA GCA GCA GGT ATC	KY_754542.1
*COX I* Reverse	CCG AAG AAT CAG AAT AGG TGT T	
*COX IV* Forward	GGC AGA ATG TTG GCT ACC	NM_017202.1
*COX IV* Reverse	GCA TAG TCT TCA CTC TTC ACA A	
*LC3b* Forward	CTT CAG GTG TGC AAT GCT GG	NM_022867.2
*LC3b* Reverse	TGG CTC TCT TCC TGT TGC TG	
*NDUFS8* Forward	AGT GTA TCT ACT GTG GTT	NM_001106322.2
*NDUFS8* Reverse	TAG CTT CTC CTT GTT GTA	
*NRF1* Forward	GGA TTC ATT ATG GCG GAA GTA A	NM_001100708.1
*NRF1* Reverse	AGT TGC TGT GGC GAG TTA	
*PGC-1α* Forward	CTG CTC TTG AGA ATG GAT ATA CTT	NM_031347.1
*PGC-1α* Reverse	CATACT TGC TCT TGG TGG AA	
*SOD2* Forward	CAC TGT GGC TGA GCT GTT GT	NM_017051.2
*SOD2* Reverse	TCC AAG CAA TTC AAG CCT CT	
*TFAM* Forward	AAA TGG CTG AAG TTG GGC GAA GTG	BC062022.1
*TFAM* Reverse	AGC TTC TTG TGC CCA ATC CCA ATG	
*18S* Forward	GTA ACC CGT TGA ACC CCA TT	NR_046239.1
*18S* Reverse	CCA TCC AAT CGG TAG TAG CG	

**Table 2:** List of antibodies used for Western blotting.

Antibody Name	Source Host	Supplier	Reference Number#
Acetylated lysines	Rabbit	Cell Signaling Technologies	9441
ADCY10/sAC	Rabbit	Abcam	Ab82854
ATP5B	Rabbit	Aviva Systems Biology	ARP48185
ATP6	Rabbit	LifeSpan BioSciences	LS-C352532
COX II	Rabbit	LifeSpan BioSciences	LS-C330986
COX IV	Rabbit	Thermo-Fisher	PA5-19471
ND5	Rabbit	LifeSpan BioSciences	LS-C368770
LC3B	Rabbit	Sigma-Aldrich	L7543
p-PKA substrates	Rabbit	Cell Signaling Technologies	9624
SIRT3	Rabbit	Cell Signaling Technologies	2627
TFAM	Rabbit	Aviva Systems Biology	ARP36993
p-threonine	Mouse	Qiagen	W10132
Biotin-XX, antimouse IgG	Goat	Thermo Fisher	W10132
Biotin-XX, antirabbit IgG	Goat	Thermo Fisher	W10142
